# Complete Genome Sequence of Biofilm-Forming Strain *Staphylococcus haemolyticus* S167

**DOI:** 10.1128/genomeA.00567-16

**Published:** 2016-06-16

**Authors:** Jisoo Hong, Jonguk Kim, Byung-Yong Kim, Jin-Woo Park, Jae-Gee Ryu, Eunjung Roh

**Affiliations:** a Microbial Safety Team, National Institute of Agricultural Sciences, Rural Development Administration, Republic of Korea; b ChunLab, Inc., Seoul National University, Seoul, Republic of Korea

## Abstract

*Staphylococcus haemolyticus* S167 has the ability to produce biofilms in large quantities. Genomic analyses revealed information on the biofilm-related genes of *S. haemolyticus* S167. Detailed studies of biofilm formation at the molecular level could provide a foundation for biofilm control research.

## GENOME ANNOUNCEMENT

*Staphylococcus haemolyticus* is the second-most frequently isolated species in human blood cultures among coagulase-negative staphylococci. *S. haemolyticus* plays an important role in nosocomial infections related to implanted medical devices, such as catheters and mechanical heart valves (1). Most urinary tract and bloodstream infections are related to indwelling medical devices, which provide suitable surfaces for biofilm formation (2). Biofilms are cooperating microbial communities attached to a surface through an extracellular polymeric matrix. Biofilms impose heavy burdens on public health (3). To investigate biofilm control strategies, complete genomic sequencing of *S. haemolyticus* S167, a strong biofilm producer, was performed.

Genome sequencing was performed using a combination of the Illumina MiSeq platform and the Pacific Biosciences (PacBio) single-molecule real-time (SMRT) sequencing platform at ChunLab (Seoul, Republic of Korea). The sequencing reads were assembled using the CLC Genomics Workbench 7.5.1 and the PacBio SMRT Analysis 2.3.0 software. Annotation of the whole-genome sequence was performed with the National Center for Biotechnology Information (NCBI) Prokaryotic Genome Automatic Annotation Pipeline (PGAP).

The complete genome of *S. haemolyticus* S167 consists of one chromosome with 2,549,338 bp (G+C content, 32.85%) and one circular plasmid with 10,808 bp (G+C content, 28.77%). The entire genome contains 2,456 protein-coding sequences (CDS), 19 rRNA genes, and 59 tRNA genes.

Genome analysis revealed that there was no *ica* operon, which has been reported to play an important role in biofilm formation in *Staphylococcus* species. Several *ica*-independent mechanisms for biofilm formation, as well as related genes, such as *bap*, *sarA*, *agr*, *fnbp*s, *aap*, *dlt*, *arlRS*, and *atl*, have been reported (4). The global regulators *agr* and *sarA*, two-component gene system *arlRS*, and the major autolysin gene *atl* were found in the sequence of *S. haemolyticus* S167. These genome analysis results provide a basis for *ica*-independent biofilm mechanism studies. A deeper understanding of biofilm formation mechanisms could provide improvement for biofilm control strategies and lead to lower medical costs.

### Nucleotide sequence accession numbers.

The complete genome sequences of the chromosome and plasmid have been deposited in the GenBank under accession numbers CP013911 and CP013912, respectively.
